# Severe and fatal interstitial lung disease induced by gemcitabine in advanced pancreatic adenocarcinoma: a case report

**DOI:** 10.1093/omcr/omad120

**Published:** 2023-11-28

**Authors:** Edwin Kelly Haag, Ganiou Adjadé, Héba Dawood, Mohammed El Fadli, Ismail Essadi, Rhizlane Belbaraka

**Affiliations:** Department of Medical Oncology, University Hospital Mohammed VI, Cadi Ayyad University, Marrakech, Morocco; Department of Medical Oncology, University Hospital Mohammed VI, Cadi Ayyad University, Marrakech, Morocco; Department of Medical Oncology, Regional Hospital of Orleans, Orleans, France; Department of Medical Oncology, Regional Hospital of Orleans, Orleans, France; Department of Medical Oncology, University Hospital Mohammed VI, Cadi Ayyad University, Marrakech, Morocco; Department of Medical Oncology, Military Hospital Avicennes, Marrakech, Morocco; Department of Medical Oncology, University Hospital Mohammed VI, Cadi Ayyad University, Marrakech, Morocco

## Abstract

Gemcitabine is a cytotoxic drug commonly used in the treatment of several types of cancer. While gemcitabine is generally considered safe and effective, it can cause some side effects, including pulmonary toxicity. Interstitial lung disease is a rare but potentially serious event. We report a case of a 63-year-old patient with advanced pancreatic adenocarcinoma. She received Gemcitabine 1000 mg/m^2^ on day 1, and day 8, and presented on day 15 of the first cycle with respiratory distress rapidly aggravating. Clinical and radiological findings were concordant with interstitial lung disease. Management consisted of high doses of corticosteroids and oxygen therapy. There was no clinical improvement and the patient passed away after a few days. Despite its low incidence, gemcitabine-induced interstitial lung disease may be responsible for a fatal clinical picture. Clinicians must be aware of this possibility and address respiratory symptoms as soon as possible.

## INTRODUCTION

Interstitial lung disease (ILD) is a heterogeneous group of conditions that cause inflammation and scarring of the lung tissue. It is characterized by a diffuse non-infectious pulmonary infiltrate on imaging, and clinical signs ranging from respiratory discomfort to a life-threatening acute respiratory distress syndrome [1].

There are multiple etiologies including drug exposure. Drug-induced ILD is an uncommon but well-recognized side effect of various medications. Antineoplastics are the agents most frequently associated with drug-induced ILD, accounting for up to 51% of cases [2], mainly bleomycin, targeted agents such as mTOR inhibitors or trastuzumab deruxtecan.

Gemcitabine is a cytotoxic drug of the pyrimidine analogs and antimetabolites family. Its safety profile is generally acceptable. Common toxicities are myelosuppression, mild nausea and vomiting, peripheral edema, and transient flu-like symptoms. Gemcitabine-induced ILD is relatively rare, with variable onset timing. In severe cases, the mortality rate can reach up to 20% [3].

In this report, we present a fatal case of diffuse ILD induced by gemcitabine in a 63-year-old woman treated for pancreatic adenocarcinoma.

## CASE REPORT

A 63-year-old woman with no particular history was diagnosed with unresectable pancreatic adenocarcinoma, following an 8-week history of abdominal pain, anorexia, fatigue, and weight loss. She had no dihydropyrimidine dehydrogenase deficiency. Initial chemotherapy with FOLFIRINOX (Fluorouracil, Leucovorin, Irinotecan, Oxaliplatin) was initiated two weeks after diagnosis. She developed a severe toxicity after the first cycle: grade III diarrhea, grade II vomiting, and acute abdominal pain, as well as painful leg swelling. Abdominal computed tomography (CT) revealed disease progression with ascites and peritoneal carcinomatosis. Compression ultrasonography with Doppler showed deep vein thrombosis of the lower extremities.

Anticoagulation with tinzaparin 8000 UI per day was initiated as well as symptomatic treatment with morphine, ondansetron, alprazolam, hydroxyzine, pantoprazole, trimebutine, phloroglucinol, furosemide, and parenteral nutrition with Smofkabiven. Upon improvement of her performance status, it was decided in the multidisciplinary discussion, to resume chemotherapy with gemcitabine monotherapy (1000 mg/m^2^ on day 1 and day 8, 21 days cycle).

**Figure 1:**
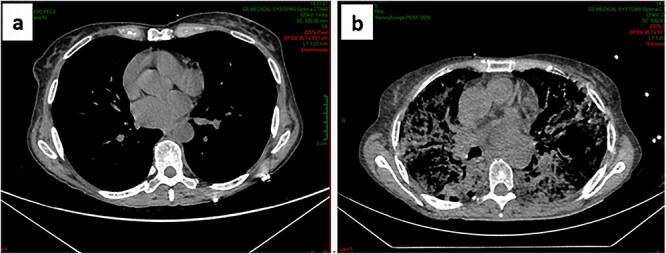
(**a**) Normal chest CT scan before the introduction of Gemcitabine; (**b**) lung scintigraphy at the onset of respiratory symptoms: diffuse bilateral infiltrate.

The patient received the first cycle (day 1 and day 8) and presented on day 15 with a rapidly aggravating dyspnea and was hospitalized. On physical examination, she had tachycardia and low oxygen saturation. She had no infectious syndrome and COVID-19 testing was negative. Laboratory evaluation showed acute renal failure. Emergency lung scintigraphy was performed. There was no sign of pulmonary embolism but a diffused bilateral interstitial infiltrate involving almost all the lung parenchyma (Fig. 1).

After pharmacological opinion, Gemcitabine involvement in the onset of pulmonary symptomatology was obvious.

**Figure 2:**
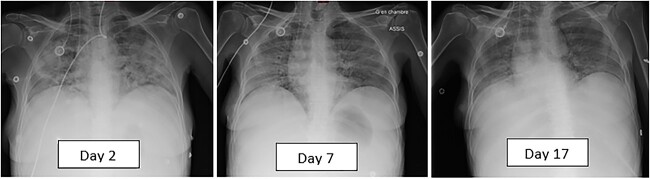
Successive chest X-rays on the second, seventh, and seventeenth day of hospitalization, showing regression of diffuse opacities with corticosteroid therapy.

The patient was managed with high doses of corticosteroid therapy (prednisone 2 mg/kg/day) and oxygen therapy by Optiflow™ at 50 l/min. Successive chest radiography showed regression of lung opacities (Fig. 2), but there was a worsening of her clinical condition. She passed away after 19 days of hospitalization.

## DISCUSSION

Several patterns of pulmonary lesions have been identified in cytotoxic adverse reactions, including acute hypersensibility, non-cardiogenic edema, interstitial infiltrate, chronic fibrosis, pleural effusion, and veno-occlusive disease [4, 5]. The mechanisms of lung lesions are not well elucidated but some hypotheses have been suggested. Gemcitabine may induce cytokines and interleukins production, in particular tumor necrosis factor-alpha (TNF-α), which are implicated in vascular lesions and lung inflammation. This hypothesis is reinforced by the response to corticosteroids [6].

A safety review of early phase II clinical trials of gemcitabine monotherapy showed that the most frequent pulmonary events are dyspnea, occurring within hours following drug administration. It is often associated with bronchospasm and rapidly resolved, rarely requiring parenteral therapy. Lung infiltrate was found in less than 1% of patients [7].

Gemcitabine-induced ILD is rare, ranging from 0.1 to 2.5%, with a median onset of 65 days according to a Japanese nationwide retrospective study [3]. Risk factors identified by the latter are age >80 years, lung cancer, and also smoking, previous chemotherapy, and late-stage cancer. Combination with other cytotoxic drugs increases the rate of lung toxicity, especially with bleomycin [8]. Gemcitabine is a known radiosensitizer, its association with radiation therapy has led to a 31.6% rate of ILD in a clinical trial [9].

Our patient presented with rapidly aggravating dyspnea and hypoxemia that needed hospitalization. Given the clinical instability and renal failure, we were not able to administrate contrast agents for computed tomography. We managed to perform lung scintigraphy which revealed diffuse interstitial pulmonary infiltration. Thereafter, the response could only be assessed with in-bed chest X-rays. According to the Common Terminology Criteria for Adverse Events (CTCAE) of drug-induced ILD, it was a grade 4 toxicity [10].

The differential diagnosis is based on the exclusion of a cardiac, infectious, metabolic cause, or carcinomatous lymphangitis [2]. The patient described had no history of cardiac disease and there was no evidence of cardiac failure or infectious disease. She had the same treatment before the introduction of gemcitabine, none of which causes pulmonary toxicity.

Treatment of ILD is based on drug cessation and corticosteroid therapy. Depending on the toxicity grade, hospitalization, and oxygen therapy may be needed [2].

Although gemcitabine-induced ILD is uncommon, it can lead to a 20% mortality rate in severe cases that need hospitalization [3]. Despite the discontinuation of the causative drug and corticosteroid administration, these patients progressively deteriorate, with severe hypoxemia requiring mechanical ventilation or even intubation. Therefore, early recognition and prompt management are critical to minimize lung damage and improve outcomes for affected patients.

## Data Availability

Data sharing is not applicable to this article as no data sets were generated or analyzed during the current study.
